# Randomised controlled trial examining the effect of exercise in people with rheumatoid arthritis taking anti-TNFα therapy medication

**DOI:** 10.1186/1471-2474-12-11

**Published:** 2011-01-13

**Authors:** Angela Reid, Audrey Brady, Catherine Blake, Anne-Barbara Mongey, Douglas J Veale, Oliver FitzGerald, Tara Cusack

**Affiliations:** 1 Physiotherapy Department, Our Lady's Hospice and Care Services, Harold's Cross, Dublin, Ireland; 2 School of Public Health, Physiotherapy and Population Science, University College Dublin, Dublin, Ireland; 3 Bone and joint Unit, St. Vincent's University Hospital, Dublin, Ireland

## Abstract

**Abstract:**

**Background**

Substantial progress has been made in the medical management of rheumatoid arthritis (RA) over the past decade with the introduction of biologic therapies, including anti-tumour necrosis factor alpha (anti-TNFα) therapy medications. However, individuals with RA taking anti-TNFα medication continue to experience physical, psychological and functional consequences, which could potentially benefit from rehabilitation. There is evidence that therapeutic exercise should be included as an intervention for people with RA, but to date there is little evidence of the benefits of therapeutic exercise for people with RA on anti-TNFα therapy medication. A protocol for a multicentre randomised controlled three-armed study which aims to examine the effect of dynamic group exercise therapy on land or in water for people with RA taking anti-TNFα therapy medication is described.

**Methods/Design:**

Six hundred and eighteen individuals with RA, on anti-TNFα therapy medication, will be randomised into one of 3 groups: a land-based exercise group; a water-based exercise group or a control group. The land and water-based groups will exercise for one hour, twice a week for eight weeks. The control group will receive no intervention and will be asked not to alter their exercise habits for the duration of the study. The primary outcome measure, the Stanford Health Assessment Questionnaire Disability Index (HAQ-DI) which measures functional ability, and secondary measures of pain, fatigue and quality of life, will be assessed at baseline, eight and 24 weeks by an independent assessor unaware of group allocation. Changes in outcome from 0 to 8 weeks and 0 to 24 weeks in the 'land-based exercise group versus control group' and the 'water-based exercise group versus control group' will be examined. Analysis will be conducted on an intention to treat basis.

**Discussion:**

This trial will evaluate the effectiveness of group exercise therapy on land or in water, for people with RA taking anti-TNFα therapy medication. If these exercise groups are found to be beneficial, they could be conducted in local community facilities thus making these forms of exercise more easily accessible for individuals and potentially reduce the burden on health services.

**Trial Registration:**

This trial is registered with ClinicalTrials.gov (a service of the United States National Institutes of Health) identifier: NCT00855322.

## Background

Rheumatoid arthritis (RA) is a chronic inflammatory, autoimmune disease that leads to progressive joint destruction and disability. It is estimated to affect in the region of 1.16% of women and 0.44% of men in the UK [1]. Arthritis Ireland reports that over 1 in 6 people currently have some form of arthritis in Ireland. It is projected that by 2031, the majority of the population will be in the 50-54 age-bracket. The incidence of arthritis increases with age and the peak incidence of conditions like RA is the 50-55 year age group. Due to this prevalence of rheumatic conditions in older people, the incidence of conditions such as RA will increase.

Substantial progress has been made in the medical management of RA over the past decade. Biologic drugs have been introduced including agents targeting tumour necrosis factor alpha (TNFα). Among them, Infliximab, Etanercept and Adalimumab have been shown in trials to reduce signs and symptoms of RA and protect joints from structural damage more effectively than conventional disease modifying anti-rheumatic drugs [2]. As no drug therapy at present leads to long-term remission for all people with RA, some people continue to experience physical, psychological and functional consequences, which could potentially benefit from rehabilitation [3]. Exercise is an important non-pharmacological therapy in RA [4].

Rheumatoid cachexia, the loss of muscle mass and strength and concomitant increase in fat mass, is very common in RA and may affect up to two-thirds of patients [5]. It is also thought to be an important contributor to increased morbidity and premature mortality in RA [6]. Rheumatoid cachexia may be attributed to high levels of TNFα [7]. There is evidence to suggest that, in the short term, anti-TNFα therapy may improve processes involved in the causation of rheumatoid cachexia, including systemic inflammation and cytokine release [8-10]. As a result of the reduced inflammatory activity and cytokinin levels in individuals with RA taking anti-TNFα medication, they may potentially be better positioned in terms of their available fat-free muscle mass to benefit from an exercise intervention than individuals not taking anti-TNFα therapy medication. It is possible that as a result of taking anti-TNFα medication, their baseline muscle quality may be better thereby increasing the effectiveness of exercise over and above that seen in individuals with RA not taking this medication. Potentially, anti-TNFα therapy medication and exercise together may be more effective than exercise alone, with the possibility that one intervention may serve to enhance the effectiveness of the other. However, to date there is little evidence in relation to the effect of exercise in people with RA currently taking anti-TNFα therapy medication.

It has recently been recommended that promotion of physical activity in older adults, and in adults aged 50 to 64 years with clinically significant chronic conditions, should emphasise moderate intensity aerobic activity, muscle strengthening activity and reducing sedentary behaviour [11]. It has been suggested that there is a case for recommendations supporting healthy physical activity behaviours among people with RA [12]. RA is associated with an increased risk of cardiovascular disease [13]. It has been recognised that women with RA are twice as likely to develop heart disease than individuals of similar age without RA, and are three times more likely to have heart disease having been diagnosed with RA for more than 10 years [14]. Therefore, general cardiovascular fitness and hence exercise participation is essential for this population.

In August 2006 an audit was undertaken of a physiotherapy review service for people with RA attending a biologic therapy outpatient clinic in a rheumatology rehabilitation unit in Dublin, Ireland. The disease status of these individuals was regarded by their consultant rheumatologists as being stable and well controlled on their anti-TNFα therapy medication. Previously these patients were not routinely reviewed by a physiotherapist, yet musculoskeletal problems still arose despite their RA being quiescent. Of the people who received physiotherapy intervention (n = 30), 70% (n = 21) required exercise prescription. This involved specific, individual exercise programmes. Group exercise programmes may represent a means of encouraging structured, ongoing exercise therapy for this group of patients.

### The evidence for land-based exercise

Exercise is considered to be an important cornerstone of the treatment of RA [15]. Exercise is physical activity that is planned, structured, and repetitive, aimed at improving or maintaining physical fitness [16]. A randomised controlled trial (RCT) by Van Den Ende et al [17] highlighted that combining intense aerobic and resistance training for individuals with RA can lead to significant enhancement in aerobic capacity and muscular strength. A trial of intensive exercise over two years by De Jong et al [18] demonstrated significant improvements in functional ability without detrimental effects on disease activity. Other randomised controlled trials support the beneficial effects of exercise in individuals with early RA with respect to muscle strength, aerobic capacity and pain, but not for functional capacity and bone mineral density [19-21]. Progressive resistance training may be an effective and safe intervention for stimulating muscle growth in people with RA [22]. Neuberger et al [23] demonstrated that symptoms associated with RA, fatigue, pain and reduced functional ability, were positively influenced by a group exercise programme.

Systematic reviews have found that aerobic and strengthening exercise lead to significant improvements in physical and psychological status without exacerbating disease activity [15,24]. The Ottawa panel concluded that good evidence exists that therapeutic exercise should be included as an intervention for people with RA [25]. Metsios et al [26] reported that there is strong evidence suggesting that exercise from low to high intensity of various modes is effective in improving disease-related characteristics and functional ability in people with RA. Guidelines for the management of RA support regular participation in dynamic and aerobic conditioning exercise programmes, to improve joint mobility, muscle strength, aerobic fitness and function, and psychological well being [27], without, in the short-term, exacerbating disease activity or joint destruction [28]. Clinical practice guidelines by Gossec et al [29] conclude that physical exercise and sports can be recommended to people with early RA and that muscle strength exercises are advisable. The European League Against Rheumatism (EULAR) recommendations for the management of early arthritis support the use of dynamic exercises as a treatment adjunct to pharmaceutical interventions [30].

In summary, therapeutic exercise has been found to be beneficial for individuals with RA, however the value of exercise for people with RA taking anti-TNFα therapy medication has yet to be established.

### The evidence for water-based exercise

Hydrotherapy involves exercising in warm water. The combined effect of the warmth and buoyancy of the water cause a reduction in joint loading, which results in an increase in ease of movement, with less pain [31]. To date, there have been few controlled trials examining hydrotherapy intervention for people with RA. Two controlled studies demonstrated that there was no negative impact on disease activity post hydrotherapy intervention [31,32]. Bilberg et al [33] found that moderate intensity pool therapy significantly improved muscle endurance in the upper and lower extremities in people with RA, but had no impact on aerobic capacity. Another RCT, carried out by Eversden et al [34], did not demonstrate any improvement in functional scores, quality of life measures or pain scores following hydrotherapy treatment. A Cochrane review of balneotherapy for people with RA, in 2003, concluded that positive findings were noted in most trials, however these should be viewed with caution due to their poor methodological quality [35]. A critical review of rehabilitation in RA similarly noted hydrotherapy to be effective, but concluded that many areas of rheumatology rehabilitation, including hydrotherapy, require more well-designed clinical trials to assess their effect more comprehensively [36]. The EULAR recommendations for the management of early arthritis support the use of hydrotherapy as a treatment adjunct to pharmacological interventions, but found insufficient evidence to support a strong recommendation [30]. In summary of the available evidence, there appears to be some positive findings to support the use of hydrotherapy, or water-based exercise, for individuals with RA. To date, there is no evidence available examining the value of hydrotherapy for individuals with RA taking anti-TNFα therapy medication.

In summary, there is evidence to support the benefits of gym and water based exercise therapy for individuals with RA, however there is no evidence evaluating these interventions for individuals with RA taking anti-TNFα medication. There is evidence to suggest that regular exercise induces anti-inflammatory effects, suggesting that physical activity per se may suppress systemic low-grade inflammation [37]. Although it has been established that anti-TNF therapy improves insulin resistance in RA, it does not seem to reverse rheumatoid cachexia [9], however it does appear that anti-TNFα therapy halts the progression of muscle deterioration. Therefore, the combination of improved muscle capacity and anti-TNFα therapy may lead to an enhanced benefit in terms of exercise participation over and above that seen in individuals with RA not taking this medication. Considering the evidence that has been presented supporting the value of exercise for individuals with RA, the addition of anti-TNFα may enhance the effectiveness of exercise prescription. By means of a RCT this study will examine the value of a prescribed gym or water based exercise programme for individuals with RA taking anti-TNFα therapy medication when compared to a control group of similar individuals receiving no exercise intervention.

## Methods/Design

### Aim

**Figure 1:**
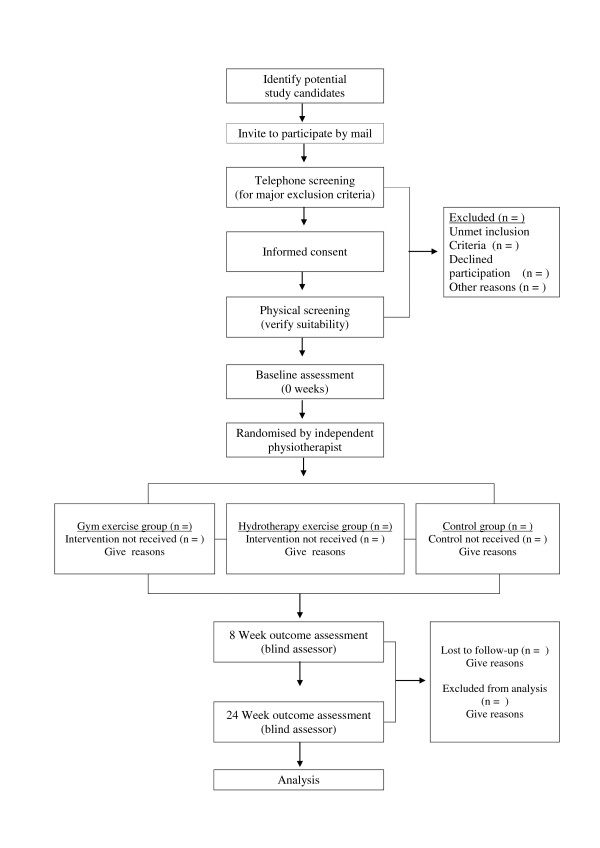
**The flow of participants through the study**.

The primary aim of this study is to examine the value of group exercise therapy for individuals with RA on anti-TNFα therapy medication. The value of land-based exercise will be compared to a control group, and the value of water-based exercise will be compared to a control group. The outcome of this study will be assessed in terms of functional ability, pain, fatigue and quality of life (QoL). Figure 1 shows the flow of participants through the study. The secondary aim of this study is to examine predictors (age, time period since diagnosis and time period on anti-TNFα therapy medication) of outcome of the Stanford Health Assessment Questionnaire Disability Index (HAQ-DI) at 8 and 24 weeks.

### Study design

A multicenter randomised controlled three-armed study will be conducted to compare the effects of group exercise (on land or in a hydrotherapy pool), to a group of participants receiving no intervention.

### Null hypothesis

People with RA taking anti-TNFα therapy medication who participate in dynamic group exercise on land or in water, will not demonstrate differences in functional ability, pain, fatigue or quality of life, when compared to a control group of individuals with RA taking anti-TNFα therapy medication who do not participate in dynamic group exercise on land or in water.

### Alternative Hypothesis

People with RA taking anti-TNFα therapy medication who participate in dynamic group exercise on land or in water, will demonstrate differences in functional ability, pain, fatigue or quality of life, when compared to a control group of individuals with RA taking anti-TNFα therapy medication who do not participate in dynamic group exercise on land or in water.

### Ethical issues

Ethical approval has been granted for this study by the St. Vincent's Healthcare Group Ethics and Medical Research Committee (Sept 2007). Licence agreements have been obtained for use of the Euroqol questionnaire in this clinical trial. This trial is registered with ClinicalTrials.gov (a service of the United States National Institutes of Health) identifier: NCT00855322.

### Study participants

A sample of people with RA taking anti-TNFα therapy medication will be recruited from a rheumatology service based in two hospitals in Dublin, Ireland.

Inclusion criteria:

• Consenting male and females, aged 18 - 70 years

• Documented diagnosis of RA as classified by the American College of Rheumatology (ACR) criteria

• Receiving anti-TNFα therapy medication for more than three months

• ACR functional class I, II, III

• Willing to exercise bi-weekly on a fixed schedule

• Living within a 50 km radius of both recruiting hospitals

Exclusion criteria:

• Physiotherapy in the previous three months

• Intra-articular corticosteroid injection within the previous four weeks

• Joint surgery in the previous six months

• Unable to understand the concepts, assessments and treatments that would be involved

• Contraindications to exercise therapy or hydrotherapy

The flow of participants through the study is presented in figure 1.

### Recruitment procedure

Possible participants will be sourced by the principal investigators (AR and AB) from a computer database of people who are currently taking anti-TNFα therapy medication and who attend a rheumatology service based in two hospitals in Dublin, Ireland. This computer database is held in St, Vincent's University Hospital. All individuals on this database, aged between 18 and 70 years, who are taking anti-TNFα therapy medication for greater than three months and who live within a 50 km radius of the recruiting hospitals, will be invited by mail to participate. A follow-up telephone call will screen for major exclusion criteria and give a detailed explanation of the study. An information leaflet/consent form will then be posted to prospective participants, where they will be asked to consider the information and give informed, written consent should they wish to participate.

### Randomisation

Participants will be randomised in predetermined block sizes by means of an allocation sequence generated using a random number table devised by a statistician using SAS software. Sealed opaque envelopes containing letters indicating group assignment will be prepared in advance using the allocation sequence. The envelopes will be drawn by an independent person not involved in the conduct of the study. Baseline assessments will be carried out by an investigator who is unaware of the allocation sequence. Following assessment, participants will be issued an envelope indicating their group allocation:

Group1 - Land-based exercise

Group 2 - Water-based exercise.

Group 3 - Control group, no intervention

### Blinding

Baseline demographics and measures of treatment outcome at baseline, eight weeks and 24 weeks will be collected by an independent assessor who is not involved in the trial and who is blinded to group allocation. Due to the nature of the exercise interventions, participants and physiotherapists conducting the exercise classes will not be blinded to treatment allocation. The primary outcome and some of the secondary outcomes, involve self-reported questionnaires being completed by participants thus precluding blinding of assessment. Participants will be asked not to divulge group allocation to the blind assessor.

### Intervention

The land- and water-based exercise groups will be supervised by the two principal investigators (AR and AB). Qualitative methods were used to determine the content and format of these exercise interventions. Interview questions were formulated to examine current expert opinion in relation to best practice. Thirteen semi-structured interviews were conducted with senior physiotherapists experienced in the areas of rheumatology and hydrotherapy. Interviews explored their expert opinions in relation to best practice. All interviews were recorded using digital recording equipment. The interviews were transcribed verbatim and analysed for emergent themes by means of a 'grounded theory' approach. Based on the results of the analysis of these interviews, appropriate gym and hydrotherapy programmes were devised. The exercise programmes were in keeping with EULAR's recommendations of dynamic exercise for the management of RA.

#### Gym Exercise Group Intervention

• Participants will attend a one hour group exercise class, twice a week for 8 weeks. This will be held in the physiotherapy gym of a rheumatology rehabilitation unit, Dublin, Ireland.

• The class will commence with 10 minutes of group warm-up exercises

• This is followed by 40 minutes of circuit-based upper/lower limb strengthening and aerobic exercises (10 stations lasting 4 minutes each)

• The class will finish with 10 minutes of cool-down exercises.

The format and content of the gym exercise intervention is detailed in Table 1.

**Table 1:** Content of the land-based (gym) exercise group

	Duration/no. of repetitions	Exercises
Warm up	5-10 mins	• Walking forwards, backwards, sideways • Joint ROM*- neck rotation/side flexion, shoulder rolls/hands behind back+ neck/shoulder abduction, elbow flexion/extension, wrist flexion/extension, trunk rotation/side flexion, hip flexion, ankle rotation • Stretches- Quad, Gastroc, Tricep, Posterior shoulder (10-15 sec hold each), latissimus dorsi stretch in supine lying

Circuit	4 mins each station	• Station 1 = Upper limb free weights (shoulder elevation, biceps curl) • Station 2 = Upper limb (wall press up, throwing ball against wall) • Station 3 = Lower limb (sit to stand, hip extension in prone lie) • Station 4 = Lower limb (single leg stands, squats) • Station 5 = Trunk (on all fours-arm/leg lifts, supine lie- leg slides) • Station 6 = Trunk ( supine lie-bridging, side lie- oyster exercise) • Station 7 = Aerobic (Stationary bike) • Station 8 = Aerobic (Walking as fast as possible) • Station 9 = Aerobic (Jogging on trampette) • Station 10 = Aerobic (Step ups)

Cool down	5-10 mins	• Walking forwards/backwards, high stepping • Posterior shoulder stretch (20 sec hold), shoulder rolls • Quads, Gastroc, Hamstring stretch (20 sec hold) • Spinal rotation, deep breathing exercises

*ROM = range of movement

#### Hydrotherapy Exercise Group Intervention

• Participants will attend a one hour group exercise class, twice weekly for 8 weeks. The class will be held in the hydrotherapy pool of a rheumatology rehabilitation unit, Dublin, Ireland.

• The class will commence with 10 minutes of group warm-up exercises.

• This will be followed by 30 minutes of group-based upper limb, lower limb and trunk flexibility/strengthening exercises. An aerobic exercise component lasting 15 minutes (5 stations lasting 3 minutes each) is circuit based.

• The class will finish with 5 minutes of cool-down exercises.

The format and content of the water-based exercise intervention is detailed in Table 2.

**Table 2:** Content of the water-based (hydrotherapy) exercise group

	Duration/no. of repetitions	Exercise
Warm up	5 mins	• Walking forwards/backwards/sideways • Jogging on spot, kicking heels to buttocks • Jumping jacks, stride jumping • Joint ROM*- neck rotation/side flexion, shoulder rolls, wrist/hand ROM, trunk rotation

Upper limb	10-20 reps	• BE* shoulder horizontal ab/adduction • BE shoulder glides into abduction • BE shoulder pro/retraction ( elbow flexion/extension) and BE shoulder extension • BA* shoulder stretch at wall into abduction and flexion (30 secs hold x 2 reps) • TR* shoulder flexion/extension, ab/adduction, horizontal ab/adduction • TR elbow flexion/extension Encouraging scapular stability and trunk stability with all upper limb exercises

Lower limb	10-20 reps	• BA hamstring stretch (30 secs hold x 2) • BA hip adductor/flexor stretch (30 sec hold × 2) • TR hip flexion/extension, ab/adduction • TR knee flexion/extension • Single leg cycling Encouraging trunk stability with all lower limb exercises

Back	10 reps	• Hanging on wall- Lumbar flexion, extension, rotation, side flexion • Standing at wall- Trunk flexion/extension • Transversus abdominus contraction while pushing float under water

Aerobic	10-15 mins (circuit - 5 stations × 2-3 mins)	• Station 1 = Aqua-jogging with running belt • Station 2 = Step ups • Station 3 = Jogging lengths of pool • Station 4 = Cycling lengths of pool sitting on woggle • Station 5 = Jogging on spot

Cool down	5 mins	• Walking forwards/backwards/sideways • Posterior shoulder stretch and tricep stretch (30 sec hold x 2) • Quads stretch (30 sec hold × 2) • Hip adductor stretch in standing- transfer weight

*ROM = range of movement, BE= buoyancy eliminated, B A = buoyancy assisted, BR = buoyancy resisted, TR= turbulence resisted

#### Control Group

• Participants will receive no intervention. They will be asked not to alter their exercise habits for the duration of the study.

### Outcome measures

At the baseline assessment, demographic information will be collected including age, gender, length of diagnosis, length of time taking anti-TNFα medication and exercise history. The assessor administering the outcomes will be blinded to participants' group allocation, and the same assessor will administer the outcomes at baseline, eight weeks and 24 weeks.

#### Primary outcome measure

• The Stanford Health Assessment Questionnaire Disability Index (HAQ-DI)

#### Secondary outcome measures

• Pain visual analogue scale- VAS

• Multidimensional Assessment of Fatigue- MAF

• Timed Chair Stand Test

• Grip Ability Test- GAT

• Fifty Foot Walk Test

• International Physical Activity Questionnaire- IPAQ short "last 7 d"

• Euroqol- EQ-5D

• Qualitative Interviews at 8 and 24 weeks

##### The Stanford Health Assessment Questionnaire Disability Index (HAQ-DI)

The HAQ-DI is the most widely used functional measure in rheumatology [38]. It was specifically developed for adults with arthritis [39]. Available evidence indicates that the HAQ-DI is feasible, reliable, valid and sensitive to change in clinical trials in RA [38].

It is a self-reported questionnaire and there are 20 items covering 8 categories. The categories are: dressing and grooming, arising, eating, walking, personal hygiene, reaching, gripping and other activities. Each item is rated from 0 to 3, with 0 = no difficulty, 1 = some difficulty, 2 = much difficulty, 3 = unable to do. The highest score within a category is used as the category score. If physical assistance or equipment is required the category score is increased to 2. The mean of the 8 categories is used to calculate the HAQ-DI score. Scores range from 0 to 3, with 0 indicating no functional disability and 3 indicating severe disability.

##### Pain Visual Analogue Scale (VAS)

This is a self-reported measure of pain severity. The pain VAS has been found to be valid, reliable and sensitive to change in non-drug clinical trials [40,41]. The scale consists of a 100 millimeter horizontal line that is anchored with verbal descriptors of "no pain" and "pain as bad as could it be" [41]. Respondents are asked to place a line perpendicular to the VAS line at the point that best indicates their pain at the present time.

##### Multidimensional Assessment of Fatigue (MAF)

The MAF is an RA-specific revision of the Piper Fatigue scale. Reasonable evidence of validation (14) and reliability [42,43] has been identified for the MAF. Sensitivity to change is shown following exercise [23]. It is a self-administered questionnaire comprising 16 questions. It measures 4 dimensions of self-reported fatigue: degree and severity, amount of distress it causes, its timing (how often it occurs and if it changed over the past week), and the degree to which it interferes with activities of daily living. Questions 1-15 form the final score (Global Fatigue Index, 0-50) while question 16 concerns change over the past week.

##### Timed Chair Stand Test

This is an objective measure of lower body strength, which is considered an indicator of functional status. It has been found to be a reasonable, reliable and valid indicator of lower body strength in generally active, community-based older adults [44]. Timed chair stand tests are found to be simple, reproducible and valid for use in an RA population [45]. The test is performed by counting the number of times an individual can stand up and sit down in 30 seconds.

##### Grip Ability Test (GAT)

This is a simple and rapid test of hand function. It was developed to evaluate hand function of people with RA [46]. The GAT is valid, reliable and sensitive to change [47]. The test consists of 3 tasks- putting a sock over one hand, putting a paper clip on an envelope and pouring water from a jug. Each task is timed and the GAT score is calculated as the sum of the weighted time for each task. A GAT score less than 20 seconds is considered normal. A high GAT score indicates decreased hand function.

##### Fifty Foot Walk Test

The 50 ft walk test is a measure of function and it is widely used in rheumatological clinical studies. It has established validity and reliability [48]. Fifty feet is marked on a flat straight floor. Participants are instructed to start walking 18 feet prior to the marked area so that stride is established prior to reaching the designated starting point. The participant is instructed to walk at their fastest pace/speed. It is measured in seconds using a stopwatch, to one hundredth of a second.

##### International Physical Activity Questionnaire (IPAQ) short "last 7 d"

The IPAQ short "last 7 d" is a self-reported measure of physical activity. It has established validity and reliability in an adult population [49]. The 9 items in the questionnaire provide information on the time spent walking, in vigorous- and moderate-intensity activity and in sedentary activity. The data gathered is used to estimate total weekly physical activity by weighting the reported minutes per week within each activity category by a Metabolic Equivalent of Task (MET) energy expenditure estimate assigned to each category. The weighted MET-minutes per week (MET^.^min^.^wk^-1^) are calculated as duration x frequency per week x MET intensity of each activity category, which are then summed.

##### Euroqol (EQ-5D)

This is a generic health status instrument designed to capture information on the overall health-related quality of life (QoL) for individuals [50]. The EQ-5D is widely used in clinical trials and has been found to be valid and reliable [51]. It consists of a questionnaire and a visual analogue scale (VAS). The questionnaire evaluates QoL in 5 dimensions - mobility, self care, usual activity, pain/discomfort and anxiety/depression. Each dimension is divided into 3 levels - no problem, some problem and extreme problem. A weighted index is used to calculate a score from 0 to 1, with 0 indicating full health and 1 indicating death. The VAS is used to evaluate a person's perception of their health state. It is scored on a scale where 0 represents the worst and 100 represents the best imaginable health state.

##### Qualitative Interviews

In the region of 50% of the exercise participants will be interviewed at 8 weeks to ascertain their perceptions of the exercise interventions. Semi-structured interviews will be conducted and recorded. They will be transcribed verbatim and analysed for emergent themes using a grounded theory approach. This affords an opportunity to capture the participants' opinion regarding their perceived benefit, satisfaction and potential compliance for continuing to attend a similar programme if available locally.

All participants who received an exercise intervention will also take part in a semi-structured interview at 24 weeks. This is to determine whether they continued to exercise following completion of the 8 week exercise programme. Potential barriers to exercise will be explored.

### Sample size considerations

The primary outcome measure (HAQ-DI) was used to calculate the sample size required. It is a widely used measure of function in rheumatological clinical trials. Previous work by Kosinski et al determined a reduction of 0.19 to be a minimally important change in the HAQ score [52] (range 0-3). Based on a two-group comparison, power calculations [53] indicated that a minimum of 171 participants per group are required to detect a reduction of 0.19 in the HAQ-DI at a two-sided significance level of 5% and a power of 80%, assuming a standard deviation of 0.63 [54]. Therefore, for this three-armed study a total of 513 participants are required. To compensate for a possible drop-out rate of 20% and to allow the sample size be divisible by three, a total sample size of 618 people is considered appropriate.

### Statistical analysis

The statistical analysis will be performed in consultation with a statistician. Statistical Package for the Social Sciences (SPSS 16) will be used to undertake the analysis. Baseline and demographic data will be presented using descriptive statistics. Differences from baseline will be calculated for all primary and secondary outcomes. Mean differences, standard deviations and 95% confidence intervals will be calculated for all continuous variables. Data will be analysed on an 'intention to treat basis' that is, all participants will be analysed in the groups to which they are randomised, whether or not they complete the intervention. Missing data will be estimated in SPSS by means of multiple imputation with a regression method.

For the primary endpoint (HAQ-DI at 8 and 24 weeks) an analysis of covariance (ANCOVA) will be used to explore between group differences in terms of the continuous variables thereby accounting for baseline scores. A preliminary examination of the data will be undertaken to ensure there is no violation of the assumptions associated with ANCOVAs. An ANCOVA adjusts each subject's follow-up score for his or her baseline score but has the advantage of being unaffected by baseline differences [55]. The fixed factor will be the group to which the participants were randomised while the covariates will be the baseline measures of the continuous outcome measures. The dependent variables will be the continuous outcome measures at 8 and 24 weeks. For secondary outcomes, if the data is not normally distributed or is categorical, the adequate tests will be employed. A significance level of 0.05 will be set for any inferential statistics conducted.

Multiple regression analysis will be used for secondary analysis in order to examine predictors of outcome at 8 and 24 weeks. A number of predictor variables age, time period since diagnosis and time period on anti-TNFα medication will be examined. The dependent variable will be dichotomous in terms of change in the HAQ (0 = change less than 0.1, 1 = change greater than or equal to 0.1). Analyses will be performed separately for the gym and hydrotherapy groups. All predictor variables will be examined for collinearity. Univariate logistic regression will be performed on all predictors thought to be associated with outcome. Those shown to be associated will be entered into the multivariate logistic regression model and backward regression will be performed. Variables with the lowest predictive value will be removed from the model if p > 0.05. Ninety five percent confidence intervals will be calculated for all final predictors.

## Conclusion

We have presented the rational and design of a randomized controlled trial to investigate the effects of participating in group exercise (on land or in water) for people with RA on anti-TNFα therapy medication, compared to non-participation. Current Irish Health Strategy proposes the provision of health services in health facilities and local communities all over Ireland. These types of exercise groups, if shown to be beneficial, are ideally suited for delivery in community settings. Treating individuals in the community will lessen the reliance on specialist services and the hospital system. This could lead to better outcomes, better health status and better cost effectiveness.

## Competing interests

The authors declare that they have no competing interests.

## Authors' contributions

AR, AB, CB and TC participated in the conception and design of this trial. AR, AB, CB and TC were responsible for writing the study protocol, devising the exercise interventions and drafting the manuscript. All authors have read and corrected draft versions of the manuscript and approved the final manuscript.

## Pre-publication history

The pre-publication history for this paper can be accessed here:

http://www.biomedcentral.com/1471-2474/12/11/prepub

## References

[B1] Symmons D Turner G Webb R Asten P Barrett E Lunt M Scott D Silman A The prevalence of rheumatoid arthritis in the United Kingdom: new estimates for a new century Rheumatology (Oxford) 2002 41 7 793 800 10.1093/rheumatology/41.7.793 12096230

[B2] Sidiropoulos PI Boumpas DT Differential drug resistance to anti-tumour necrosis factor agents in rheumatoid arthritis Ann Rheum Dis 2006 65 6 701 703 10.1136/ard.2005.049890 16699049 PMC1798160

[B3] Vliet Vlieland TP Rehabilitation of people with rheumatoid arthritis Best Pract Res Clin Rheumatol 2003 17 5 847 861 10.1016/S1521-6942(03)00043-3 12915161

[B4] Vliet Vlieland TP Pattison D Non-drug therapies in early rheumatoid arthritis Best Pract Res Clin Rheumatol 2009 23 1 103 116 10.1016/j.berh.2008.08.004 19233050

[B5] Engvall IL Elkan AC Tengstrand B Cederholm T Brismar K Hafstrom I Cachexia in rheumatoid arthritis is associated with inflammatory activity, physical disability, and low bioavailable insulin-like growth factor Scand J Rheumatol 2008 37 5 321 328 10.1080/03009740802055984 18666027

[B6] Roubenoff R Rheumatoid cachexia: a complication of rheumatoid arthritis moves into the 21st century Arthritis Res Ther 2009 11 2 108 10.1186/ar2658 19439037 PMC2688195

[B7] Roubenoff R Roubenoff RA Cannon JG Kehayias JJ Zhuang H Dawson-Hughes B Dinarello CA Rosenberg IH Rheumatoid cachexia: cytokine-driven hypermetabolism accompanying reduced body cell mass in chronic inflammation J Clin Invest 1994 93 6 2379 2386 10.1172/JCI117244 8200971 PMC294444

[B8] Rall LC Roubenoff R Rheumatoid cachexia: metabolic abnormalities, mechanisms and interventions Rheumatology (Oxford) 2004 43 10 1219 1223 10.1093/rheumatology/keh321 15292530

[B9] Marcora SM Chester KR Mittal G Lemmey AB Maddison PJ Randomized phase 2 trial of anti-tumor necrosis factor therapy for cachexia in patients with early rheumatoid arthritis Am J Clin Nutr 2006 84 6 1463 1472 17158431 10.1093/ajcn/84.6.1463

[B10] Metsios GS Stavropoulos-Kalinoglou A Douglas KM Koutedakis Y Nevill AM Panoulas VF Kita M Kitas GD Blockade of tumour necrosis factor-alpha in rheumatoid arthritis: effects on components of rheumatoid cachexia Rheumatology (Oxford) 2007 46 12 1824 1827 10.1093/rheumatology/kem291 18032540

[B11] Nelson ME Rejeski WJ Blair SN Duncan PW Judge JO King AC Macera CA Castaneda-Sceppa C Physical activity and public health in older adults: recommendation from the American College of Sports Medicine and the American Heart Association Circulation 2007 116 9 1094 1105 10.1161/CIRCULATIONAHA.107.185650 17671236

[B12] Eurenius E Stenstrom CH Physical activity, physical fitness, and general health perception among individuals with rheumatoid arthritis Arthritis Rheum 2005 53 1 48 55 10.1002/art.20924 15696555

[B13] Solomon DH Goodson NJ Katz JN Weinblatt ME Avorn J Setoguchi S Canning C Schneeweiss S Patterns of cardiovascular risk in rheumatoid arthritis Ann Rheum Dis 2006 65 12 1608 1612 10.1136/ard.2005.050377 16793844 PMC1798453

[B14] Solomon DH Karlson EW Rimm EB Cannuscio CC Mandl LA Manson JE Stampfer MJ Curhan GC Cardiovascular morbidity and mortality in women diagnosed with rheumatoid arthritis Circulation 2003 107 9 1303 1307 10.1161/01.CIR.0000054612.26458.B2 12628952

[B15] Van den Ende CH Vliet Vlieland TP Munneke M Hazes JM WITHDRAWN: Dynamic exercise therapy for treating rheumatoid arthritis Cochrane Database Syst Rev 2008 1 CD000322 18253972 10.1002/14651858.CD000322.pub2 PMC10798406

[B16] Caspersen CJ Powell KE Christenson GM Physical activity, exercise, and physical fitness: definitions and distinctions for health-related research Public Health Rep 1985 100 2 126 131 3920711 PMC1424733

[B17] van den Ende CH Breedveld FC le Cessie S Dijkmans BA de Mug AW Hazes JM Effect of intensive exercise on patients with active rheumatoid arthritis: a randomised clinical trial Ann Rheum Dis 2000 59 8 615 621 10.1136/ard.59.8.615 10913058 PMC1753212

[B18] de Jong Z Munneke M Zwinderman AH Kroon HM Jansen A Ronday KH van Schaardenburg D Dijkmans BA Van den Ende CH Breedveld FC Is a long-term high-intensity exercise program effective and safe in patients with rheumatoid arthritis? Results of a randomized controlled trial Arthritis Rheum 2003 48 9 2415 2424 10.1002/art.11216 13130460

[B19] Hakkinen A Sokka T Kotaniemi A Kautiainen H Jappinen I Laitinen L Hannonen P Dynamic strength training in patients with early rheumatoid arthritis increases muscle strength but not bone mineral density J Rheumatol 1999 26 6 1257 1263 10381039

[B20] Hakkinen A Sokka T Kotaniemi A Hannonen P A randomized two-year study of the effects of dynamic strength training on muscle strength, disease activity, functional capacity, and bone mineral density in early rheumatoid arthritis Arthritis Rheum 2001 44 3 515 522 10.1002/1529-0131(200103)44:3<515::AID-ANR98>3.0.CO;2-5 11263764

[B21] Hakkinen A Sokka T Hannonen P A home-based two-year strength training period in early rheumatoid arthritis led to good long-term compliance: a five-year followup Arthritis Rheum 2004 51 1 56 62 10.1002/art.20088 14872456

[B22] Marcora SM Lemmey AB Maddison PJ Can progressive resistance training reverse cachexia in patients with rheumatoid arthritis? Results of a pilot study J Rheumatol 2005 32 6 1031 1039 15940763

[B23] Neuberger GB Press AN Lindsley HB Hinton R Cagle PE Carlson K Scott S Dahl J Kramer B Effects of exercise on fatigue, aerobic fitness, and disease activity measures in persons with rheumatoid arthritis Res Nurs Health 1997 20 3 195 204 10.1002/(SICI)1098-240X(199706)20:3<195::AID-NUR3>3.0.CO;2-D 9179174

[B24] Stenstrom CH Minor MA Evidence for the benefit of aerobic and strengthening exercise in rheumatoid arthritis Arthritis Rheum 2003 49 3 428 434 10.1002/art.11051 12794800

[B25] Panel Ottawa Ottawa Panel evidence-based clinical practice guidelines for therapeutic exercises in the management of rheumatoid arthritis in adults Phys Ther 2004 84 10 934 972 15449978

[B26] Metsios GS Stavropoulos-Kalinoglou A Veldhuijzen van Zanten JJ Treharne GJ Panoulas VF Douglas KM Koutedakis Y Kitas GD Rheumatoid arthritis, cardiovascular disease and physical exercise: a systematic review Rheumatology (Oxford) 2008 47 3 239 248 10.1093/rheumatology/kem260 18045810

[B27] American College of Rheumatology Guidelines for the management of rheumatoid arthritis: 2002 Update Arthritis Rheum 2002 46 2 328 346 10.1002/art.10148 11840435

[B28] Luqmani R Hennell S Estrach C Birrell F Bosworth A Davenport G Fokke C Goodson N Jeffreson P Lamb E British Society for Rheumatology and british health professionals in Rheumatology guideline for the management of rheumatoid arthritis (the first two years) Rheumatology (Oxford) 2006 45 9 1167 1169 10.1093/rheumatology/kel215a 16844700

[B29] Gossec L Pavy S Pham T Constantin A Poiraudeau S Combe B Flipo RM Goupille P Le Loet X Mariette X Nonpharmacological treatments in early rheumatoid arthritis: clinical practice guidelines based on published evidence and expert opinion Joint Bone Spine 2006 73 4 396 402 10.1016/j.jbspin.2006.01.008 16626995

[B30] Combe B Landewe R Lukas C Bolosiu HD Breedveld F Dougados M Emery P Ferraccioli G Hazes JM Klareskog L EULAR recommendations for the management of early arthritis: report of a task force of the European Standing Committee for International Clinical Studies Including Therapeutics (ESCISIT) Ann Rheum Dis 2007 66 1 34 45 10.1136/ard.2005.044354 16396980 PMC1798412

[B31] Hall J Skevington SM Maddison PJ Chapman K A randomized and controlled trial of hydrotherapy in rheumatoid arthritis Arthritis Care Res 1996 9 3 206 215 10.1002/1529-0131(199606)9:3<206::AID-ANR1790090309>3.0.CO;2-J 8971230

[B32] Suomi R Collier D Effects of arthritis exercise programs on functional fitness and perceived activities of daily living measures in older adults with arthritis Arch Phys Med Rehabil 2003 84 11 1589 1594 10.1053/S0003-9993(03)00278-8 14639556

[B33] Bilberg A Ahlmen M Mannerkorpi K Moderately intensive exercise in a temperate pool for patients with rheumatoid arthritis: a randomized controlled study Rheumatology (Oxford) 2005 44 4 502 508 10.1093/rheumatology/keh528 15728422

[B34] Eversden L Maggs F Nightingale P Jobanputra P A pragmatic randomised controlled trial of hydrotherapy and land exercises on overall well being and quality of life in rheumatoid arthritis BMC Musculoskelet Disord 2007 8 23 10.1186/1471-2474-8-23 17331241 PMC1821024

[B35] Verhagen AP Bierma-Zeinstra SM Cardoso JR de Bie RA Boers M de Vet HC Balneotherapy for rheumatoid arthritis Cochrane Database Syst Rev 2003 4 CD000518 14583923 10.1002/14651858.CD000518

[B36] Hammond A Rehabilitation in rheumatoid arthritis: a critical review Musculoskeletal Care 2004 2 3 135 151 10.1002/msc.66 17041978

[B37] Brandt C Pedersen BK The role of exercise-induced myokines in muscle homeostasis and the defense against chronic diseases J Biomed Biotechnol 2010 520258 20224659 10.1155/2010/520258 PMC2836182

[B38] Patricia P Katz for the Association of Rheumatology Health Professionals Outcomes Measures Task Force Measures of adult general functional status: The Barthel Index, Katz Index of Activities of Daily Living, Health Assessment Questionnaire (HAQ), MACTAR Patient Preference Disability Questionnaire, and Modified Health Assessment Questionnaire (MHAQ) Arthritis Care & Research 2003 49 S5 S15 S27

[B39] Fries JF Spitz P Kraines RG Holman HR Measurement of patient outcome in arthritis Arthritis Rheum 1980 23 2 137 145 10.1002/art.1780230202 7362664

[B40] Ferraz MB Quaresma MR Aquino LR Atra E Tugwell P Goldsmith CH Reliability of pain scales in the assessment of literate and illiterate patients with rheumatoid arthritis J Rheumatol 1990 17 8 1022 1024 2213777

[B41] Carol S Burckhardt KDJ Adult measures of pain: The McGill Pain Questionnaire (MPQ), Rheumatoid Arthritis Pain Scale (RAPS), Short-Form McGill Pain Questionnaire (SF-MPQ), Verbal Descriptive Scale (VDS), Visual Analog Scale (VAS), and West Haven-Yale Multidisciplinary Pain Inventory (WHYMPI) Arthritis Care & Research 2003 49 S5 S96 S104

[B42] Belza BL Henke CJ Yelin EH Epstein WV Gilliss CL Correlates of fatigue in older adults with rheumatoid arthritis Nurs Res 1993 42 2 93 99 10.1097/00006199-199303000-00006 8455994

[B43] Belza BL Comparison of self-reported fatigue in rheumatoid arthritis and controls J Rheumatol 1995 22 4 639 643 7791155

[B44] Jones CJ Rikli RE Beam WC A 30-s chair-stand test as a measure of lower body strength in community-residing older adults Res Q Exerc Sport 1999 70 2 113 119 10380242 10.1080/02701367.1999.10608028

[B45] Newcomer KL Krug HE Mahowald ML Validity and reliability of the timed-stands test for patients with rheumatoid arthritis and other chronic diseases J Rheumatol 1993 20 1 21 27 8441160

[B46] Dellhag B Bjelle A A Grip Ability Test for use in rheumatology practice J Rheumatol 1995 22 8 1559 1565 7473483

[B47] Dellhag B Bjelle A A five-year followup of hand function and activities of daily living in rheumatoid arthritis patients Arthritis Care Res 1999 12 1 33 41 10.1002/1529-0131(199902)12:1<33::AID-ART6>3.0.CO;2-4 10513488

[B48] Marks R Reliability and validity of self-paced walking time measures for knee osteoarthritis Arthritis Care Res 1994 7 1 50 53 10.1002/art.1790070111 7918728

[B49] Craig CL Marshall AL Sjostrom M Bauman AE Booth ML Ainsworth BE Pratt M Ekelund U Yngve A Sallis JF International physical activity questionnaire: 12-country reliability and validity Med Sci Sports Exerc 2003 35 8 1381 1395 10.1249/01.MSS.0000078924.61453.FB 12900694

[B50] The EuroQol Group EuroQol--a new facility for the measurement of health-related quality of life Health Policy 1990 16 3 199 208 10.1016/0168-8510(90)90421-9 10109801

[B51] Alison Carr Adult Measures of Quality of Life: The Arthritis Impact Measurement Scales (AIMS/AIMS2), Disease Repercussion Profile (DRP), EuroQoL, Nottingham Health Profile (NHP), Patient Generated Index (PGI), Quality of Well-Being Scale (QWB), RAQoL, Short Form-36 (SF-36), Sickness Impact Profile (SIP), SIP-RA, and World Health Organization's Quality of Life Instruments (WHOQoL, WHOQoL-100, WHOQoL-Bref) Arthritis Care & Research 2003 49 S5 S113 S133

[B52] Kosinski M Zhao SZ Dedhiya S Osterhaus JT Ware JE Jr Determining minimally important changes in generic and disease-specific health-related quality of life questionnaires in clinical trials of rheumatoid arthritis Arthritis Rheum 2000 43 7 1478 1487 10.1002/1529-0131(200007)43:7<1478::AID-ANR10>3.0.CO;2-M 10902749

[B53] Daly LE Bourke GJ Interpretation and Uses of Medical Statistics 2000 Dublin: Blackwell Scientific Publications

[B54] Wells G Li T Maxwell L MacLean R Tugwell P Determining the minimal clinically important differences in activity, fatigue, and sleep quality in patients with rheumatoid arthritis J Rheumatol 2007 34 2 280 289 17304654

[B55] Vickers AJ Altman DG Statistics notes: Analysing controlled trials with baseline and follow up measurements BMJ 2001 323 7321 1123 1124 10.1136/bmj.323.7321.1123 11701584 PMC1121605

